# Supplementary material for: A Critical View of the Application of the APEX Software (Aqueous Photochemistry of Environmentally-Occurring Xenobiotics) to Predict Photoreaction Kinetics in Surface Freshwaters
Source: Molecules. 2019 Dec 18;25(1):9. doi: 10.3390/molecules25010009 (PMC7017383; doi:10.3390/molecules25010009)
Supplement: Supplementary file 1 [file molecules-25-00009-s001.zip › 3.2.4_gcc-4.4.0.zip!/3.2.4_gcc-4.4.0/3.2.4_gcc-4.4.0/doc/octave/html/interpreter/Distribution.html]

Distribution - Untitled


Previous: How You Can Contribute to Octave,
Up: Preface


---

### Distribution

Octave is free software. This means that everyone is free to
use it and free to redistribute it on certain conditions. Octave
is not, however, in the public domain. It is copyrighted and there are
restrictions on its distribution, but the restrictions are designed to
ensure that others will have the same freedom to use and redistribute
Octave that you have. The precise conditions can be found in the
GNU General Public License that comes with Octave and that also appears
in Copying.

Octave is available on CD-ROM, with various collections of other free
software, from the Free Software Foundation. Ordering a copy of
Octave from the Free Software Foundation helps to fund the development
of more free software. For more information, write to
> Free Software Foundation  
> 51 Franklin Street, Fifth Floor  
> Boston, MA 02110-1301–1307  
> USA

Octave can also be downloaded from http://www.octave.org, where
additional information is available.
